# Editorial: Identification and Characterization of Contrasting Genotypes/Cultivars to Discover Novel Players in Crop Responses to Abiotic/Biotic Stresses

**DOI:** 10.3389/fpls.2021.784874

**Published:** 2021-11-17

**Authors:** Raul A. Sperotto, Felipe K. Ricachenevsky, Elizabeth R. Waters, Guihua Bai, Magdalena Arasimowicz-Jelonek

**Affiliations:** ^1^Graduate Program in Biotechnology, Life Sciences Area, University of Taquari Valley–Univates, Lajeado, Brazil; ^2^Graduate Program in Cell and Molecular Biology, Botany Department, Federal University of Rio Grande Do Sul, Porto Alegre, Brazil; ^3^Department of Biology, San Diego State University, San Diego, CA, United States; ^4^USDA-ARS Hard Winter Wheat Genetics Research Unit, Manhattan, KS, United States; ^5^Department of Plant Ecophysiology, Faculty of Biology, Adam Mickiewicz University, Poznań, Poland

**Keywords:** drought, temperature, salinity, nutrition, toxic metals, biotic factors

Plants are continuously exposed to several abiotic and biotic stresses that negatively affect crop performance and yield. It is predicted that climate change will enhance the frequency and extent of such negative impacts on crop yield (Chadalavada et al., 2021). Continued greenhouse gas emissions will cause further rise in temperature, leading to increased evapotranspiration and drought severity, soil salinity, and local temperature extremes that reduce plants' capability to defend against insects and pathogenic microorganisms (Baillo et al., 2019). Thus, multifactorial stresses constitute one of the most pressing threats in agricultural areas worldwide (Bai et al., 2018; Saijo and Loo, 2020). This has raised a major challenge for plant scientists to secure global food supply and brought an immediate need to continuously increase the yield of major food crops. Understanding stress-related processes in plants is crucial to develop screening procedures for selecting crop lines that can better adapt to non-optimal conditions. One of the most common strategies used by plant scientists is to identify and characterize genotypes/cultivars with contrasting phenotypes and differential responses to the non-optimal conditions, and find molecular, biochemical and physiological mechanisms involved in crop responses to abiotic and biotic stresses (Nutan et al., 2018; Xu and Bassel, 2020). The elucidation of such mechanisms will facilitate the identification and further characterization of agronomically important genes, alleles, and molecules for creating/engineering new cultivars to improve crop yield under stress. This Research Topic provides an update on recent advances in the plant molecular and physiological mechanisms associated with plant responses to stressful conditions, and provides an overview of different approaches used for improving crop tolerance/resistance. Here, we highlight some of the major points arising from these reports.

## Abiotic Stress Responses

Climate change is expected to intensify stressful conditions to most crops, which will limit their yield potential and threaten global food supply (Chadalavada et al., 2021). Several studies demonstrated that some genotypes are more tolerant to non-optimal conditions than others. However, the mechanisms explaining variation in plant tolerance have not been well-characterized in most crops. In this section, we show that several studies were conducted in different crops to identify new genetic resources and tools for the characterization of the possible mechanisms of plant responses and tolerance to abiotic stresses.

### Drought Stress Response

Using artificial mutagenesis, le Roux et al. characterized a wheat mutant line tolerant to drought stress at late booting stage, and found that such mutant line maintained higher relative moisture and photosynthesis rate, besides producing more seeds than its WT progenitor under drought stress. The mutant also showed increased expression of RuBisCO and a set of unique proteins associated with dehydration tolerance, which are probably involved in wheat drought tolerance. Terminal drought is the most significant abiotic stress affecting wheat grain yields worldwide. Figueroa-Bustos et al. investigated the effects of root system size on terminal drought and grain yield by comparing the responses of two genotypes with different root sizes under terminal drought treatments, and found that “Tincurrin” genotype (with smaller root system size) showed less reduction in leaf stomatal conductance, photosynthesis and transpiration rates, and thus higher water use efficiency and grain yield than “Bahatans-87” (with larger root size). Faster phenological development and quick grain filling avoided severe water stress in “Tincurrin”. In other cereal crops, Degraeve et al. explored the use of vulnerability to *drought-induced xylem embolism* (DIXE) as a proxy for drought tolerance. The acoustic emissions (AE_50_) values suggested the wheat cultivar “Excalibur” as the most tolerant, and the triticale cultivar “Dublet” as the most vulnerable to DIXE, though “Dublet” had significantly higher hydraulic capacitances that are essential for internal water storage to temporarily buffer water shortage. In addition, both cultivars have a contrasting trade-off between hydraulic safety and efficiency. They suggested that both AE_50_ and hydraulic capacitances should be considered when evaluating a cultivar's drought responses.

Root pressure has been associated with increased crop production under drought. Drobnitch et al. observed that root pressure was triggered by drought exposure followed by re-watering in *Sorghum bicolor* and was positively associated with fine/coarse root length ratio and shoot biomass. Root allocation may have a possible role in creating root pressure and adaptive benefit of root pressure for shoot biomass production. Through RNA-seq they identified different expression of aquaporins, membrane transporters and ATPases in the plants with root pressure.

To select drought tolerant potato lines, Haas et al. developed a model based on leaf metabolite and transcripts to predict drought tolerance of potato lines in breeding programs. After testing the model in multiple environments, they found that it effectively selected superior drought tolerant genotypes, but not low tolerant or sensitive genotypes. They recommend using the model as an alternative tool to yield-based selection in arid environments by combining with tools that can select against sensitive genotypes.

In soybean, wild relatives may carry novel QTLs for drought avoidance. Prince et al. developed an inter-specific mapping population between cultivated and wild soybean and characterized the population for root architecture traits. Using a high-density SNP map, they identified 11 QTLs for these traits. Two of them on chromosome 7A of wild soybean were significant for total root length (RL) and root surface area (RSA). Four candidate genes were identified for the RL QTLs. The novel QTLs from the wild soybean could be used to improve root system architecture and productivity of elite soybean lines under drought stress.

Kamphorst et al. evaluated the performance of field cultivated popcorn inbred lines under well-watered and water stressed (ψ_soil_≥ −1.5 MPa) conditions. The tolerant lines maintained a stay-green condition (higher SPAD index) until physiological maturity. Besides leaf greenness, traits related to leaf photosynthetic status and stomatal conductance are good indicators of the agronomic performance of popcorn under water constraint.

Cao et al. investigated the drought tolerance mechanisms of mulberry by comparing physiological responses and transcriptional changes of plasma membrane intrinsic proteins (PIPs) in different plant tissues of three cultivars with contrasting drought tolerance under progressive soil water deficit. They found that in the resistant cultivar “Wubu”, whole-plant level responses of *PIP* transcription, osmoregulation through proline and soluble proteins, and antioxidative protection are important mechanisms for mulberry to cope with drought stress.

Alvarez-Maldini et al. investigated differences in morpho-physiological traits of native *Cryptocarya alba* and *Persea lingue* from central Chile under water stress and found that the most mesic populations showed more reduction in relative growth rates than the xeric populations in response to water stress in both species. Also, they found that water use efficiency (WUE) in *C. alba* and the two traits (WUE and relative growth rates) in *P. lingue* could further assist in the selection of populations for restoration according to their response to water stress.

Gedam et al. screened 100 onion genotypes for drought tolerance using multivariate analysis. Bulb yield was strongly positively correlated with membrane stability index, relative water content, total chlorophyll content, antioxidant enzyme activity, and leaf area under drought stress. Therefore, the identified tolerant genotypes with drought-adaptive traits may be useful in onion breeding program for drought tolerance and can significantly improve onion productivity in low rainfall areas.

Simulating drought stress in natural field conditions, Arif et al. evaluated the drought tolerance of advanced breeding lines of chickpea. Environment influenced seed yield and number of branches, pods, and seeds, while genotype was the main source of variation in seed weight and plant height. Some genotypes performed well only in non-stressed environments, while others performed consistently well in both environments. Ranks in each environment and genotype selection index will help breeders to select genotypes of their choice for further use as variety or pre-breeding germplasm.

Olive plants growing in the greenhouse were evaluated for drought tolerance under severe stress. Baccari et al. analyzed growth, plant water status, net photosynthesis rates, chlorophyll contents and the extent of photo- and antioxidant defenses, and concluded that “Zarrazi” and “Chemlali” genotypes showed better performance under severe water stress compared to the other genotypes, which was associated to their ability to trigger a higher antioxidant protection. Also, olive plants were able to continue photosynthesizing under severe stress, which is probably related to increased levels of xanthophyll cycle de-epoxidation and α-tocopherol contents.

Harb et al. evaluated the transcriptomic profile of drought-tolerant “Otis” and drought-sensitive “Baronesse” barley genotypes subjected to drought. “Otis” had better photosynthetic capacity under drought compared to “Baronesse”, which could be attributed to the differences in the expression level of photosystem II proteins D1 and D2, both associated with PSII stability. Also, many potential drought tolerance genes such as wax biosynthesis gene (*CER1*), and two *NAC* TFs were uniquely induced in “Otis” under drought stress. Therefore, the overall transcriptional responses were low in “Otis”, but the genes that could confer drought tolerance were either specifically induced or greatly upregulated in this genotype.

Mechanisms of lignin distribution regarding cell wall composition and digestibility under contrasted water regimes were studied in maize internodes from a recombinant inbred line (RIL) population grown in field trials (El Hage et al.). Biochemical and histological traits have different response thresholds to water deficit. While histological profiles were only modified under pronounced water deficit, most of the biochemical traits responded whatever the strength of the water deficit. The authors also highlight the potential role of *p*-coumaroylation of maize stem in refining plant agronomic properties and digestibility.

Using high throughput phenomics facility, Lekshmy et al. demonstrated a significant genetic variability in the acquired tolerance to a gradually imposed drought stress protocol among contrasting rice genotypes. Association of free radical production/scavenging with yield attributes showed that genotypes that were able to maintain low levels of free radicals in their cells presented high spikelet fertility and hence grain yield under drought stress, evidencing the importance of assessing genetic variability to improve drought adaptation.

### Cold Stress Response

Yang et al. performed high-throughput QTL-sequencing analyses to identify the major QTLs governing cold tolerance at the bud-bursting (CTBB) stage in rice. They found a novel major QTL (*qCTBB9*) on chromosome 9 that controls seed survival rate under low-temperature conditions. Re-sequencing data and local QTL mapping allowed mapping *qCTBB9* to a region containing 58 annotated genes, but only one (Os09g0444200) was strongly induced by cold stress. Therefore, Os09g0444200 is a potential candidate for *qCTBB9* and could be used to improve the cold tolerance of rice varieties by marker-assisted selection.

Zhang et al. found that membrane lipid and fatty acid metabolism may be a significant contributor in peanut cold tolerance. Lipidomic data indicated that photosynthetic damage, resulted from the alteration in fluidity and integrity of photosynthetic membranes under cold stress, were mainly caused by decreased monogalactosyldiacylglycerol levels, and could be relieved by increased digalactosyldiacylglycerol and sulfoquinovosyldiacylglycerol levels, maintaining chloroplast structural integrity and normal photosynthesis. The upregulation of phosphatidate phosphatase and phosphatidate cytidylyltransferase under cold stress inhibited the damage of membrane lipid peroxidation caused by excessive accumulation of phosphatidic acid.

Photosynthetic efficiency is a key determinant and a good indicator of cold tolerance in plants. Huang et al. used associative transcriptomics to identify genetic loci controlling photosynthetic gas exchange parameters in 123 rapeseed (*Brassica napus* L.) accessions. Twenty two candidate genes were identified, and *Cab026133.1* encoding a tropinone reductase (*BnTR1*) was further confirmed to be closely linked to the transpiration rate. Overexpressing *BnTR1* in Arabidopsis plants increased the transpiration rate and the alkaloids content, resulting in enhanced cold tolerance under freezing conditions.

### Heat Stress Response

In their study, Wang et al. examined the impact of heat-stress at three developmental stages in two varieties of *Zea mays* contrasting to heat response. It was shown that heat stress has significant impacts on development/morphology in both varieties. However, significant differences among the two varieties were found in hormone pathways. After exposure to heat stress, some but not all hormone pathways respond the same way in the two varieties, such as the upregulation of genes for gibberellic acid and abscisic acid, and the downregulation of those for zeatin and zeatin riboside. In contrast, the genes involved in salicylic acid (SA) and jasmonic acid (JA) pathways are upregulated in the heat-tolerant variety and downregulated in the heat-sensitive variety.

Lu et al. characterized near-isogenic lines (NIL) from two populations of wheat (Cascades X Tevere; Cascades X W165) and identified four pairs of NILs that varied in heat responses. Genotyping identified five single nucleotide polymorphisms (SNPs) in seven candidate genes in a major heat tolerance QTL on chromosome 7A. The genes identified code for the following proteins: a zinc finger domain, an F-box domain, a galactose oxidase, a peroxidase, a sugar transporter, and an HSP-70 protein. In another study of wheat, Tomás et al. examined four Portuguese landraces for heat-tolerant traits. Specifically, they examined the impact of heat on grain yield and protein content. They report that heat stress did impact grain composition, and, importantly, that the impact of heat stress varied across the landraces. This variability supports the use of these genetic resources to increase wheat tolerance to heat stress.

Chaudhary et al. present a review of the traits associated with heat tolerance within a wide range of crop species. The authors distinguish between growth-based parameters, leaf traits, pollen-based traits, biochemical traits, differential gene expression, and yield traits. This analysis provides an opportunity to identify common pathways and processes that control heat tolerance.

Most heat-stress studies examine the impact of daytime stress. This is due to many reasons, including the well-established impact of heat stress on photosynthesis. Huang et al. make an important contribution by examining the impact of nighttime temperatures on yield in rice. These authors used long-term climate data to evaluate the impact of high nighttime temperatures on both a hybrid cultivar and an inbred cultivar. Notably, this study clearly shows that nighttime temperatures can have significant impacts on yield. Further, they demonstrate that these impacts can vary across genetic backgrounds.

### Salt Stress Response

Gil-Muñoz et al. examined salt tolerance in four distinct populations of Persimmon (*Diospyros*). The open-pollinated *D. virginiana* possessed the highest level of salt tolerance. In contrast, the *D. lotus* seedlings had the lowest tolerance to salinity. Principal component analysis of the morphological responses to salinity had the widest distribution in the *D. virginiana* populations and the lowest in the *D. lotus* populations. These data indicate the presence of significant variation within this genus for salinity tolerance. This in turn suggests that future breeding programs have sufficient genetic variation to successfully breed salt-tolerant hybrid persimmon varieties.

The responses to salt stress in two cultivars of sweet potato, Xushu 22 (salt-tolerant) and Xushu 32 (salt-sensitive), were examined by Meng et al.. During stress, the salt-sensitive cultivar differentially expressed twice as many genes as did the tolerant variety. It is also reported that the genes upregulated in the tolerant and sensitive cultivars vary. The more tolerant cultivar upregulated genes associated with the ribosome pathway, as well as genes associated with glucosinolate, and N-Glycan biosynthesis.

Niron et al. examined the response to salt stress in tolerant and susceptible genotypes of common bean (*Phaseolus vulgaris*). The Ispir genotype (tolerant) differentially expressed more genes than did the TR43477 genotype (susceptible). Genes associated with photosynthesis, porphyrin, and chlorophyll were enriched during stress in Ispir but not in TR43477. In addition, sucrose and starch metabolism were enriched in Ispir but declined in TR43477. Ispir was also able to better regulate ion content including sodium (Na^+^) compared to TR43477. Finally, amino acid metabolism was normal in Ispir but dysregulated during salt stress in TR43477.

### Combined Responses to Abiotic Stresses

Studies focused on abiotic stress responses commonly isolate stresses to understand their physiological and molecular underpinnings. However, these stresses often occur in a combination with the environments, and therefore studies focused in plant responses to combined abiotic stresses are very important. Dubberstein et al. explored how single and combined heat and drought stresses can affect photosynthesis in two genotypes of *Coffea* sp., namely Icatu and CL153. Icatu was clearly more drought-tolerant, while both genotypes were impacted by high temperature and a combination of both stresses. Authors showed that photochemical components are resilient to single and combined stresses, while enzymes involved in photosynthesis are more sensitive, suggesting a pathway for improving *Coffea* sp. tolerance to environmental changes. Other two studies investigated heat and drought responses in wheat plants. El Habti et al. explored how water use and carbohydrate partitioning changed in eight bread wheat (*Triticum aestivum* L.) that vary in stress tolerance. The final grain yield was associated with total water use and aboveground biomass as stresses were more intense. Importantly, glucose and fructose measurements in grains 12 days after anthesis explained more than 40% of the variation in grain weight in the main spike. Authors also found that older wheat varieties increased soluble carbohydrate partitioning from stem to spike when stressed, while modern varieties did not show the same behavior. Moreover, it was clear that maintaining transpiration was key for stress tolerance and productivity, especially when heat and drought were combined. Xue et al. investigated the role of *TaPYL4* gene (involved in plant responses to a variety of stresses) in regulating development of major agronomic traits in wheat under multiple environments (well-watered, drought and heat stress treatments). Functional markers were developed for *TaPYL4-2A* and *TaPYL4-2B* haplotypes, and used to scan a natural population of common wheat accessions for correlation analysis between genotypes and phenotypic traits. *TaPYL4-2A* and *TaPYL4-2B* were identified to be significantly correlated with plant growth-related traits, showing that *TaPYL4* could be a valuable target gene in wheat genetic improvement to develop the ideal plant architecture.

Heat and drought affect grapevine (*Vitis* sp.) cultivation worldwide. Carvalho et al. identified tolerant genotypes within 255 clones of the Aragonez (syn. Trempanillo) variety, commonly grown in Portugal and Spain, using leaf temperature of plants exposed to both stresses. Analyses were performed in 3 years and combined with must characteristics for ranking tolerant genotypes that maintained yield and quality. Selected genotypes with contrasting tolerance/sensitivity to stresses were also evaluated for marker gene expression, identifying selection markers or possible targets for stress tolerance improvement. With that, authors were able to find tolerant clones that maintain must quality.

Wang et al. described the GRAS transcription factor gene family in soybean (*Glycine max*). Authors identified 117 GRAS genes in soybean genome, which were classified in subfamilies and were shown to be regulated by drought, salinity, abscisic acid (ABA) and brassinosteroids (BR). Authors selected *GmGRAS37* for functional characterization. Ectopic expression of *GmGRAS37* in soybean hairy roots leads to up-regulation of several stress-related genes, and to increased salt and drought tolerance. This work provides an excellent candidate gene for soybean improvement and a comprehensive description of the GRAS gene family in this crop.

### Nutritional Stress Response

Nutritional stress is a major problem for agricultural systems worldwide. Fertilization is a necessary cost of production, but can lead to environmental pollution, whereas toxic elements found in soils can decrease plant growth or end up in edible organs such as the seeds. Arsenic (As) is a toxic element that can accumulate in rice grains. As accumulation in the segregating population derived from Lemont X TeQing crosses was evaluated by Fernández-Baca et al. when plants were grown under different wetting/drying regimes, a common practice in rice field. It was found that severe but not mild wetting/drying cycles can reduce inorganic As (the most toxic form) accumulation both in the field and greenhouse conditions. Seven QTLs associated with As accumulation were identified, suggesting that combination of genotypes and water management practices can effectively reduce As in rice grains.

García de la Torre et al. performed a screening on 258 *Medicago truncatula* accessions for cadmium (Cd) tolerance using relative root growth as a proxy to identify tolerant and sensitive genotypes. Cd or other elements concentration in plant tissues were not correlated with tolerance. Authors suggested that NADPH recycling enzymes might be associated with tolerance, while increased expression of enzymes involved in glutathione and phytochelatin synthesis might not be a good marker for Cd tolerance in *M. truncatula*. In another study concerning toxic elements to plants, Ambachew and Blair used a common bean (*Phaseolus vulgaris*) Andean diversity panel composed of 227 genotypes to search for variation in aluminum (Al) tolerance. Genome-wide association using root traits suggested known transporters involved in secretion of malate and citrate as candidate genes, suggesting that increased organic acid secretion is the basis for Al tolerance in this common bean genetic pool.

White Guinea yam (*Discorea rotundata*) tubers are an important crop in West Africa, especially to resource-poor farmers. Matsumoto et al. investigated 20 yam breeding lines and one local variety for their ability to grow in nutrient-poor soils and for responses to added fertilizers. Authors identified tolerant and susceptible genotypes and suggest that biomass vigor may be a marker to select high yield yams. The work provides evidence for genotypic variation for tolerance to low nutrient in yam, which can be used for breeding more resilient varieties. Also focusing on nutritional deficiency, Oustric et al. evaluated combinations of scion and rootstocks of clementine (*Citrus clementina* Hort. ex Tan). Authors identified scion/rootstock combinations that were less affected by nutritional deficiency, but also found that using tetraploid rootstocks does not improve tolerance compared to the same diploid genotype.

### Other Stresses Responses

Urban et al. presents the proteome response of two genotypes (Cadeli and Viking) of winter oilseed rape microspore-derived embryos (MDE) to PEG-induced osmotic stress. The approach revealed that osmotic resistance is manifested by the accumulation of proteins engaged in energy metabolism, redox homeostasis, protein destination, and signaling including phospholipases and MAPK4. Additional analysis of hormonal profiles during the stress progression showed that the osmotic resistance noted in cultivar Cadeli was also associated with the time-dependent accumulation of ABA, SA, JA, gibberellin, as well as active cytokinins.

## Biotic Stress Responses

Biotic stress causes immense damage to agricultural products worldwide and raises the risk of hunger in many areas (Moustafa-Farag et al., 2019). In this section, we highlight six studies conducted in four different crops (rice, tomato, melon, and apple) that help to elucidate the plant responses and resistance mechanisms to bacteria, fungi, viruses, thrips, and mites.

Kumar et al. summarize the molecular mechanisms associated with the interaction between rice and *Xanthomonas oryzae* pv. *oryzae* responsible for bacterial blight disease. The authors underline that the use of genetic and genomic tools allowed to reveal genetic background of resistance to bacterial blight in rice, and at least 44 genes conferring resistance were identified. Out of these genes, at least 11 have been cloned and characterized favoring molecular breeding, as many resistant rice cultivars and hybrids have been developed and released worldwide. Buffon et al. presented the responses of various rice species/cultivars to *Schizotetranychus oryzae* mite infestation. Surprisingly, the resistant response to phytophagous mites was noted only during *Oryza sativa* cv. Nipponbare-*S. oryzae* interaction, while *Oryza barthii* presented high sensitivity to the mites. Under infested conditions, Nipponbare presented higher protein abundance associated with detoxification, higher levels of proline and a greater abundance of defense-related proteins, such as osmotin, ricin B-like lectin, and protease inhibitors.

Marchant et al. evaluated whether continuous use of tomato yellow leaf curl virus (TYLCV) resistant cultivars promotes overcoming TYLCV resistance and/or affects TYLCV regional diversity. Examination of the full-length genomes of naturally occurring TYLCV isolates from TYLCV-resistant and susceptible tomato genotypes indicated that the genomes did not differentiate with increasing transmission number, as well as no specific mutations were repeatedly observed, and no positive selection was detected. Therefore, tomato resistance might not be exerting selection pressure against TYLCV to facilitate development of resistance-breaking strains. According to Mouden et al., tomato seed treated with JA reduces feeding damage provoked by western flower thrips. Importantly, the effect seems to be cultivar-dependent, and tomato seed coat permeability was indicated as a key factor for effective JA absorption and induction of JA-mediated thrips defense. The results may be particularly valuable for the seed industry to perform pre-treatments on non-responsive cultivars, as well as for tomato breeding programs to select for genetic traits that affect seed permeability.

By the combined application of bulked segregant analysis (BSA) with RNAseq, Cao et al. detected a novel QTL (*qCmPMR-12*) for melon resistance to powdery mildew. Additionally, RNAseq analysis indicated that the *MELO3C002434* gene encoding an ankyrin repeat-containing protein is the most likely candidate gene associated with the resistance to the powdery mildew, caused by *Podosphaera xanthii*. Moreover, the authors developed 15 suitable KASP markers for breeding.

Using six apple rootstock genotypes with contrasting phenotypes to *Pythium ultimum* infection, Zhu et al. identified major miRNA pathways and candidate genes involved in apple root defense activation. Their findings suggest that quick and enhanced lignification of apple roots by the action of laccase may significantly impede pathogen penetration and minimize the disruption of effective defense activation in roots of resistant genotypes.

## Combined Abiotic and Biotic Stress Responses

In the natural environment, plants are constantly subjected to a combination of abiotic and biotic stresses simultaneously. It is critical to understand how the various response pathways to these stresses interact with one another within the plants, and where the points of crosstalk occur which switch the responses from one pathway to another (Ku et al., 2018). Two articles fall into this category of combined abiotic and biotic stress responses.

Li, Wang et al. performed transcriptome studies proving that *GhTLPs* (thaumatin-like proteins) participate in cotton responses to both stress and developmental stimuli. At physiological level, silencing of *GhTLP19* in cotton was associated with increased content of malondialdehyde and reduced catalase activity, besides increasing the sensitivity to *Verticillium dahliae* and drought. A contrasting response (as higher proline content, thicker and longer trichomes, and higher drought tolerance) was shown by Arabidopsis plants overexpressing *GhTLP19*.

Li, Xin et al. identified a new aspect of pesticide metabolism in cucumber plants, as propamocarb is commonly used to prevent plants against downy mildew. It was found that *CsHMGB* gene contributes to lower propamocarb residue levels in cucumber, and functional analyses revealed that overexpression of *CsHMGB* in D9320 genotype, which naturally accumulates high level of propamocarb, could alleviate the pesticide phytotoxicity. The effect of phytotoxicity mitigation was manifested by an increase in the waxiness of the cuticle and stomatal conductance in the pericarp cells, as well as improved potential of antioxidant system.

## Final Comment

In summary, the work presented here documents recent advances in the plant responses to non-optimal conditions. Now, the challenge is to move forward, to integrate this information and to develop knowledge-driven tolerance/resistance strategies against a diverse range of stressful conditions.

## Author Contributions

All authors listed have made a substantial, direct and intellectual contribution to the work, and approved it for publication.

## Conflict of Interest

The authors declare that the research was conducted in the absence of any commercial or financial relationships that could be construed as a potential conflict of interest.

## Publisher's Note

All claims expressed in this article are solely those of the authors and do not necessarily represent those of their affiliated organizations, or those of the publisher, the editors and the reviewers. Any product that may be evaluated in this article, or claim that may be made by its manufacturer, is not guaranteed or endorsed by the publisher.
